# The Association Between Mechanical Power Within the First 24 Hours and ICU Mortality in Mechanically Ventilated Adult Patients With Acute Hypoxemic Respiratory Failure: A Registry-Based Cohort Study

**DOI:** 10.1016/j.chest.2025.03.012

**Published:** 2025-03-28

**Authors:** Stephan von Düring, Kuan Liu, Laveena Munshi, S. Joseph Kim, Martin Urner, Neill K.J. Adhikari, Ken Kuljit S. Parhar, Eddy Fan

**Affiliations:** a Division of Intensive Care Medicine, Department of Acute Care Medicine, Geneva University Hospitals, Geneva, Switzerland; b Department of Anaesthesiology, Pharmacology, Intensive Care and Emergency Medicine, Faculty of Medicine, University of Geneva, Geneva, Switzerland; c Interdepartmental Division of Critical Care Medicine, University of Toronto, Toronto, ON, Canada; d Institute of Health Policy, Management and Evaluation, University of Toronto, Toronto, ON, Canada; e Department of Medicine, University of Toronto, Toronto, ON, Canada; f Department of Anesthesiology & Pain Medicine, University of Toronto, Toronto, ON, Canada; g Department of Critical Care Medicine, Sunnybrook Health Sciences Centre, Toronto, ON, Canada; h Division of Nephrology, University Health Network, Toronto, ON, Canada; i Division of Respirology, Department of Medicine, University Health Network, Toronto, ON, Canada; j Toronto General Hospital Research Institute, Toronto, ON, Canada; k Department of Critical Care Medicine, Alberta Health Services, Calgary, AB, Canada; l O’Brien Institute for Public Health, University of Calgary, Calgary, AB, Canada; m Libin Cardiovascular Institute, University of Calgary, Calgary, AB, Canada

**Keywords:** positive-pressure respiration, pulmonary ventilation, respiratory distress syndrome, respiratory insufficiency, ventilator-induced lung injury

## Abstract

**Background:**

Despite the widespread adoption of lung-protective ventilation strategies, mortality among patients receiving invasive mechanical ventilation (IMV) remains high. Mechanical power (MP) integrates various variables responsible for ventilator-induced lung injury and has been associated with mortality in patients with ARDS. However, the impact of MP on ICU mortality in the larger group of patients with acute hypoxemic respiratory failure (AHRF) has not been well established, and previous studies have reported inconsistent thresholds for predicting outcomes.

**Research Question:**

Is high MP (> 17 J/min) within the first 24 hours of IMV, calculated using dynamic driving pressure, associated with ICU mortality in patients with AHRF? Additionally, does a threshold exist below which IMV is considered safe?

**Study Design and Methods:**

In this multicenter cohort study, we included adult patients with AHRF who received IMV. Patients were excluded if they received IMV for > 24 hours before inclusion or were receiving extracorporeal life support. We applied multivariable logistic regression models with inverse probability of treatment weighting and used change-point regression models with restricted cubic splines.

**Results:**

Of the 21,714 patients in our registry, 9,031 patients (42%) met the inclusion criteria. After adjusting for baseline characteristics, high MP was associated with increased ICU mortality (OR, 1.58; 95% CI, 1.44-1.72), with a nonlinear dose-response relationship. No consistent safe MP threshold was identified. High MP also was associated with lower extubation rates and fewer ventilator-free days.

**Interpretation:**

In this study, exposure to high MP within the first 24 hours of IMV was associated with increased ICU mortality in patients with AHRF. The absence of a consistent safe threshold suggests that reducing MP at IMV initiation may be a strategy to improve outcomes, warranting exploration in clinical trials.

## Graphical Abstract

**Figure:**
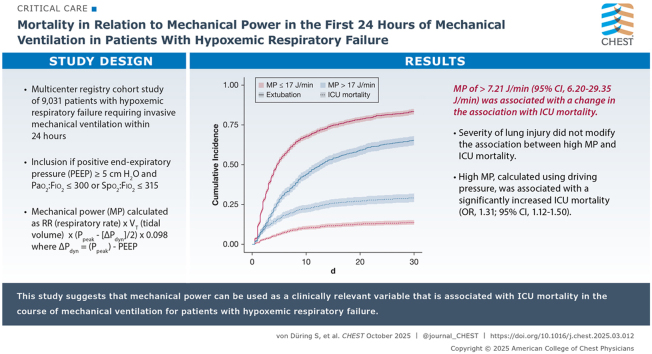

FOR EDITORIAL COMMENT, SEE PAGE 843

**Take-Home Points**

**Study Question:** Is high mechanical power (MP; > 17 J/min) within the first 24 hours of invasive mechanical ventilation (IMV), calculated using dynamic driving pressure, associated with increased ICU mortality in patients with acute hypoxemic respiratory failure, and does a threshold exist below which MP is considered safe?

**Results:** High MP within the first 24 hours was associated with increased ICU mortality (OR, 1.58; 95% CI, 1.44-1.72), but no definitive safe MP threshold was identified.

**Interpretation:** The association between high MP and increased ICU mortality, coupled with the lack of a consistent threshold, suggests that reducing MP at the initiation of IMV may improve outcomes, meriting further investigation in clinical trials.

Invasive mechanical ventilation (IMV) is a lifesaving intervention in the ICU.^1^ Despite lung-protective ventilation strategies, mortality rates remain high, prompting the exploration of novel approaches.^2^, ^3^, ^4^, ^5^ Strategies such as low tidal volume (V_T_) and pressure limitation have been established to decrease ventilator-induced lung injury (VILI) and mortality.^6^^,^^7^ Recent evidence suggests that these benefits may extend beyond ARDS to the broader category of acute hypoxemic respiratory failure (AHRF).^8^^,^^9^

VILI, a serious complication of IMV, results in acute damage or exacerbation of preexisting lung injury.^10^, ^11^, ^12^ Each ventilator-delivered breath requires energy to overcome the resistive and elastic forces of the respiratory system.^13^^,^^14^ This energy is not recovered completely during exhalation, resulting in its absorption and dissipation as kinetic energy within the respiratory system.^13^ Excessive energy is thought to have deleterious effects on the extracellular matrix, alveolar cells, and lung capillaries, contributing to VILI.^15^ This is particularly important because patients with AHRF are especially prone to sustaining additional lung damage associated with IMV.^16^

Recent studies have quantified the energy transferred by the ventilator to the respiratory system. Mechanical power (MP) represents the inflation energy transferred by the ventilator to the respiratory system per minute and encompasses all parameters thought to be associated with the genesis of VILI, including volume, pressure, flow, resistance, and respiratory rate.^17^^,^^18^ Several studies have demonstrated an association between MP and patient outcomes in patients with and without ARDS,^19^, ^20^, ^21^, ^22^, ^23^, ^24^ including those with acute brain injury,^25^ reinforcing its relevance across all patients receiving IMV. A recent study determined that an MP of > 17 J/min during the second 24 hours of IMV was associated with increased mortality in patients with ARDS, suggesting that maintaining MP below this threshold could represent a new target for lung-protective ventilation.^19^

Current lung-protective strategies use static measurements obtained through manual flow pause, allowing for respiratory system relaxation and redistribution of volume and distending pressures. However, static measurements may underestimate the maximum pressure experienced by vulnerable lung units during dynamic inflation in heterogeneous lungs.^26^ Additionally, static measurements used for calculating MP (eg, plateau pressure) are infrequent and typically are reserved for severely ill patients requiring significant respiratory support and receiving deep sedation, neuromuscular blockade, or both, thereby limiting MP evaluation to a specific subset of patients already at increased risk of VILI.^2^^,^^27^ Moreover, current methods for MP calculation rely on the absence of spontaneous breathing efforts. By using dynamic driving pressure (ΔP_dyn_), MP may provide a more feasible estimate of VILI risk at the bedside, is available at each breath, and is applicable to all ventilation modes.^28^, ^29^, ^30^, ^31^ However, the ability of baseline MP, calculated using ΔP_dyn_, to predict patient outcomes in the ICU remains uncertain.

Our primary objective was to estimate the association between high (> 17 J/min) and low (≤ 17 J/min) MP within the first 24 hours of IMV, calculated using ΔP_dyn_, and ICU mortality in adult patients with AHRF. The secondary objective was to explore the existence of a safe MP threshold that could serve as a potential clinical target to improve lung-protective ventilation.

## Study Design and Methods

### Data Source and Study Population

This multicenter registry-based cohort study used data from the Toronto Intensive Care Observational Registry. Data were collected daily at 8 am in 9 ICUs affiliated with the University of Toronto between April 2014 and January 2023. We included adult patients (aged ≥ 18 years) receiving IMV with a positive end-expiratory pressure (PEEP) of ≥ 5 cm H_2_O and AHRF, defined as Pao_2_ to Fio_2_ ratio of ≤ 300 mm Hg or percentage of oxygen in the blood to Fio_2_ ratio of ≤ 315 after ICU admission. Patients receiving IMV for > 24 hours before inclusion or receiving extracorporeal life support were excluded. Ethics approval was obtained from the research ethics board of the University of Toronto (RIS human protocol no.: 43926).

### Exposure and Outcomes of Interest

MP was calculated within the first 24 hours of IMV, representing the initial lung parameters closest to the time of IMV initiation. As carried out previously, MP was calculated as respiratory rate × V_T_ × (peak pressure [P_peak_] – (ΔP_dyn_) / 2) × 0.098, where ΔP_dyn_ represents P_peak_ – PEEP.^28^, ^29^, ^30^, ^31^ MP was categorized as high (> 17 J/min) or low (≤ 17 J/min) for the primary objective. This threshold was chosen because it has been reported in several studies^19^^,^^32^^,^^33^ and lies between thresholds reported in other studies.^20^^,^^21^ MP also was modeled continuously for our secondary objective. Our primary outcome was ICU mortality. Secondary outcomes included duration of IMV (accounting for the competing risk of death), ventilator-free days (VFDs) at 28 days, extent of nonrespiratory organ failure at 48 hours (as measured by the nonpulmonary Sequential Organ Failure Assessment [SOFA] score^34^), severity of shock at 48 hours (as measured by the administration of norepinephrine equivalents^35^), and markers of VILI (as measured by the incidence of chest tubes during the first 7 days, serving as an indicator of pneumothoraces requiring decompression and potentially attributed to high-intensity IMV).

### Statistical Analysis

Baseline variables are summarized using descriptive statistics for the overall population and were stratified by high and low MP. Continuous variables are presented as median (interquartile range), and categorical variables are presented as total numbers and percentages.

In the primary analysis, we estimated the effect of exposure to high MP on ICU mortality. To address potential confounding in the absence of randomization, we used a propensity score (PS)-based inverse probability of treatment weighting approach between groups.^36^ The PS was estimated from a multivariable logistic regression model with 23 clinically relevant covariates, encompassing potential confounders known to be associated with the outcome (e-Fig 1). We assessed the balance of baseline characteristics between MP groups before and after PS weighting using standardized differences, with values exceeding 0.2 indicative of covariate imbalance after weighting.^37^ The OR quantifying the association between MP and mortality with 95% CI then was estimated with a second multivariable logistic regression model using the inverse probability of treatment weighting samples. Based on prior knowledge and clinical expertise, we adjusted for the following confounders in our directed acyclic graph: age, sex, BMI, severity of illness (Acute Physiology and Chronic Health Evaluation III and SOFA scores), Pao_2_ to Fio_2_ ratio, and arterial pH (e-Fig 2). To account for missing data at baseline, we performed multiple imputation by chained equations and generated 5 imputed data sets for the full study population. Details on missing data patterns and handling procedures are provided in e-Tables 1, 2.

Several secondary analyses were performed. First, we used multivariable logistic regression models, adjusting for the confounders in our directed acyclic graph, to compare the conditional treatment effect with the marginal treatment effect from our PS model. Second, through a change-point analysis, we sought to explore the existence of an MP threshold beyond which was associated a change in ICU mortality. To characterize the association, we initially compared 2 regression models: a standard logistic regression with a linearly specified relationship between MP and the log odds of ICU mortality, and a logistic regression with a restricted cubic spline. Both were adjusted for confounders in our directed acyclic graph, and model comparison was based on Akaike information criterion and concordance statistic for goodness-of-fit comparison. The model deemed best fit then was used to conduct a single-threshold change-point regression analysis to identify an MP level associated with a significant change in ICU mortality.^38^ Third, we used a Fine and Gray subdistribution hazard model to estimate the probability of successful IMV liberation between MP groups, accounting for death as a competing risk and censoring beyond 30 days. Gray’s K-sample test compared cumulative incidence functions. Fourth, VFDs during the first 28 days were defined as the number of days alive and free of IMV for > 48 consecutive hours. The association between MP and VFDs was assessed using a multivariable linear regression model, treating VFDs as a continuous outcome. Fifth, through multivariable linear regressions, we assessed the association between MP groups on disease severity at 48 hours (nonpulmonary SOFA score or norepinephrine equivalent), adjusting for disease severity at baseline. Finally, we applied a negative binomial regression model to assess the cumulative count of chest tubes placed within the first 7 days across MP groups, accounting for the count-based nature of this outcome with consideration of data overdispersion. To address missing data in the complete-case data set for both disease severity at 48 hours and chest tube placement, we used inverse probability weighting, with weights derived from a logistic regression model predicting missingness.

### Subgroup and Sensitivity Analyses

Several sensitivity analyses were conducted to test the strength of the main results. First, we repeated the analyses using complete-case data to evaluate the robustness of the findings in our primary objective. Second, we conducted a cutoff analysis in which MP was partitioned into 10 groups (deciles). This partitioning allowed us to determine the cutoff point beyond which the association between MP and the odds of ICU mortality became positive when compared with the median MP of the entire cohort.^19^ Third, to detect the presence of effect modification, we measured the effect of IMV between MP groups on ICU mortality, stratified by severity of lung injury (Pao_2_ to Fio_2_ ratio, ventilatory ratio, and respiratory system elastance normalized to predicted body weight), while accounting for the risk of α inflation by applying a Bonferroni correction when possible.^39^ Fourth, to assess the potential impact of unmeasured confounding on the association between MP and ICU mortality, we calculated an E-value.^40^ Finally, we applied regression models to a subcohort of patients with controlled modes of ventilation, where driving pressure (ΔP) was calculated as plateau pressure minus PEEP.

Additional methodologic details are provided in e-Appendix 1. Statistical significance was defined as a 2-sided *P* value of < .05, and all analyses were conducted using R version 4.3.0 (R Foundation for Statistical Computing). Reporting followed the Strengthening the Reporting of Observational Studies in Epidemiology Statement recommendations.

## Results

### Primary Outcome

**Figure 1:**
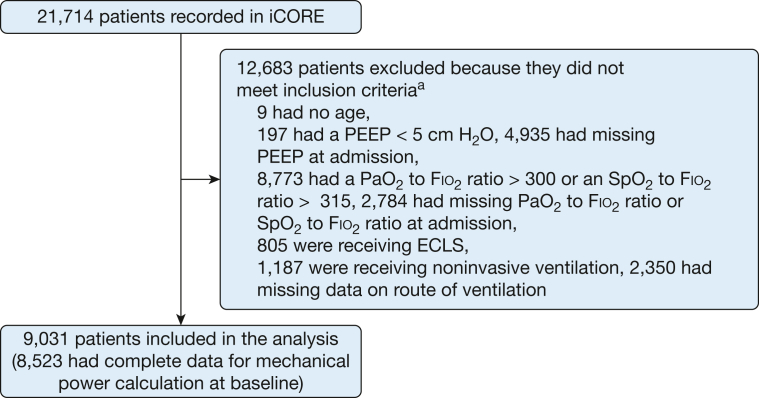
Study flowchart showing that among the 21,714 patients initially recorded in our registry, 9,031 patients met the inclusion criteria and subsequently were included in our analysis. iCORE = Toronto Intensive Care Observational Registry; ECLS = extracorporeal life support; PEEP = positive end-expiratory pressure; Spo_2_ = percentage of oxygen in the blood. ^a^Patients could fulfill multiple exclusion criteria.

**Figure 2:**
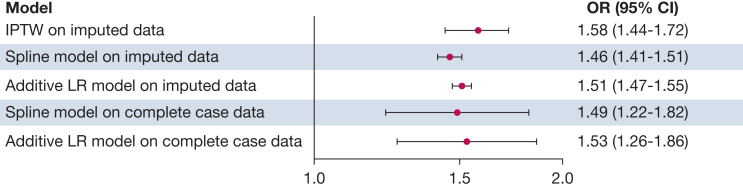
Forest plot showing the association between baseline mechanical power (MP) of > 17 J/min and ICU mortality. The OR represents the odds of ICU mortality when MP was > 17 J/min. Sensitivity analyses were performed to determine whether results were dependent on method of covariate adjustment or imputation. Complete case data, n = 3,421 patients after adjusting. Imputed data, m = 5, n = 9,031 patients. IPTW = inverse probability of treatment weighting; LR = logistic regression.

**Figure 3:**
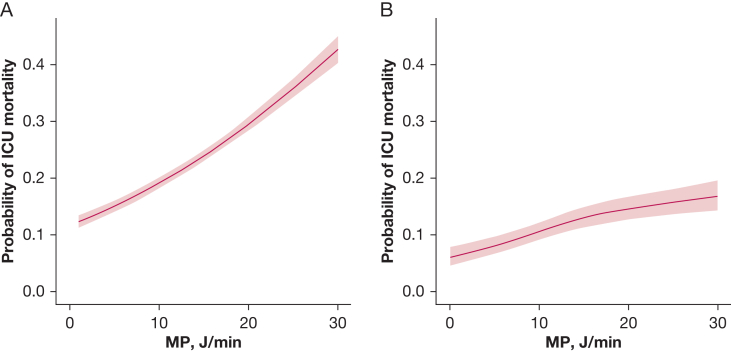
A, B, Line graphs showing the association between baseline mechanical power of ≤ 30 J/min at baseline and the probability of ICU mortality using the first copy of the imputed data set: unadjusted linear regression model with only the intercept as a predictor variable (A) and multivariable restricted cubic spline regression model adjusting for confounders in our directed acyclic graph (B). MP, mechanical power.

Of the 21,714 patients enrolled in the registry, 12,683 patients (58%) did not meet our inclusion criteria by the time of data collection (had resolved AHRF, were discharged, or had missing data), and 805 patients were receiving extracorporeal life support at baseline (Fig 1). Our analysis focused on 9,031 patients with a median age of 62 years (interquartile range, 50-72 years), and 3,374 patients (37%) were women (Table 1). High MP at baseline was associated with increased ICU mortality (OR, 1.58; 95% CI, 1.44-1.72) (Fig 2). This was consistent with results from our unweighted multivariable logistic regression models. A restricted cubic spline model best fit our data (e-Table 3). We observed a nonlinear relationship between MP and ICU mortality where the probability of mortality rose with increasing MP (Fig 3, e-Figs 3-5). Our change-point analysis identified that an MP of > 7.21 J/min (95% CI, 6.20-29.35 J/min) was associated with a change in the association with ICU mortality (e-Table 4).

**Table 1:** Baseline Characteristics of Patients With Acute Hypoxemic Respiratory Failure and Receiving Invasive Mechanical Ventilation

Characteristic	Overall (N = 9,031)	MP, J/min^a^		SMD
		≤ 17 (n = 5,960)	> 17 (n = 2,563)	
Baseline characteristics and severity of illness				
Age, y	62 (50-72)	63 (52-73)	60 (48-71)	0.14
Female sex	3,374 (37)	2,449 (41)	742 (29)	0.26
BMI, kg/m^2^	27 (23-32)	27 (23-31)	28 (24-34)	0.25
APACHE III score	76 (57-96)	73 (54-91)	85 (67-108)	0.45
SOFA score	6 (3-9)	5 (3-8)	7 (5-10)	0.51
Pao_2_ to Fio_2_ ratio	222 (155-287)	244 (183-297)	171 (117-243)	0.59
Spo_2_ to Fio_2_ ratio	227 (166-247)	240 (194-250)	178 (125-226)	0.85
Ventilatory ratio	1.55 (1.25-1.99)	1.38 (1.14-1.68)	2.00 (1.64-2.48)	1.10
Comorbidities at baseline				
Cardiovascular disease	3,037 (34)	2,055 (34)	826 (32)	0.05
Respiratory disease	2,206 (24)	1,507 (25)	626 (24)	0.02
Diabetes	2,098 (23)	1,320 (22)	660 (26)	0.08
Active neoplasm	825 (9.1)	536 (9.0)	235 (9.2)	0.01
Chronic kidney disease	777 (8.6)	474 (8.0)	255 (9.9)	0.07
Immunosupression and AIDS	429 (4.8)	268 (4.5)	145 (5.7)	0.05
Liver failure	624 (6.9)	416 (7.0)	195 (7.6)	0.02
Reason for mechanical ventilation				
Acute respiratory failure	6,285 (70)	3,961 (66)	1,946 (76)	0.21
Altered level of consciousness	3,009 (33)	2,125 (36)	723 (28)	0.16
Acute CPD exacerbation	207 (2.3)	127 (2.1)	76 (3.0)	0.05
Neuromuscular causes	50 (0.6)	38 (0.6)	9 (0.4)	0.04
Not available	244 (2.7)	189 (3.2)	54 (2.1)	0.07
Blood gasses				
Spo_2_, %	97 (95-99)	97 (95-99)	97 (94-98)	0.17
Pao_2_, mm Hg	96 (79-127)	97 (80-129)	93 (75-122)	0.14
Paco_2_, mm Hg	40 (35-46)	39 (35-45)	40 (35-48)	0.12
Arterial pH	7.35 (7.29-7.41)	7.37 (7.31-7.42)	7.32 (7.24-7.38)	0.49
Ventilator settings				
Pressure-control ventilation, %	3,154 (35)	2,059 (35)	1,066 (42)	0.15
Volume-control ventilation, %	2,834 (31)	1,290 (22)	1,096 (43)	0.46
Pressure-support ventilation, %	2,788 (31)	2,459 (41)	302 (12)	0.71
Other methods of ventilation, %	75 (0.8)	45 (0.8)	26 (1.0)	0.03
Fio_2_, %	40 (40-55)	40 (40-50)	52 (40-70)	0.79
V_T_ by PBW, mL/kg^b^	6.6 (6.0-7.9)	6.7 (5.9-8.0)	6.6 (6.0-7.8)	0.03
Respiratory rate, breaths/min^c^	22 (18-27)	20 (16-23)	28 (24-32)	1.40
PEEP, cm H_2_O^d^	8 (5-10)	5 (5-8)	10 (8-12)	1.20
Plateau pressure, cm H_2_O^e^	19 (15-23)	17 (14-20)	22 (18-26)	0.91
Peak inspiratory pressure, cm H_2_O^f^	22 (17-27)	19 (15-23)	29 (25-34)	1.60
MP, J/min^a^	13 (9-18)	10 (8-13)	22 (19-27)	2.50

Data are presented as No. (%) or median (interquartile range) unless otherwise indicated. Baseline data were collected at 8 am within the first 24 hours of invasive mechanical ventilation. Mechanical power was calculated using dynamic driving pressure. Other methods of ventilation included pressure-regulated volume-control, proportional assist ventilation, neurally adjusted ventilatory assist, synchronized intermittent mandatory ventilation, and volume support ventilation. APACHE = Acute Physiology and Chronic Health Evaluation; CPD = chronic pulmonary disease; MP = mechanical power; PBW = predicted body weight; PEEP = positive end-expiratory pressure; SMD = standardized mean difference; SOFA = Sequential Organ Failure Assessment; Spo_2_ = percentage of oxygen in the blood; V_T_ = tidal volume.

a Data available for 8,523 patients (94%).

b Data available for 5,962 patients (66%).

c Data available for 9,019 patients (99%).

d Data available for 9,031 patients (100%).

e Data available for 1,093 patients (12%).

f Data available for 8,583 patients (95%).

### Secondary Outcomes

**Figure 4:**
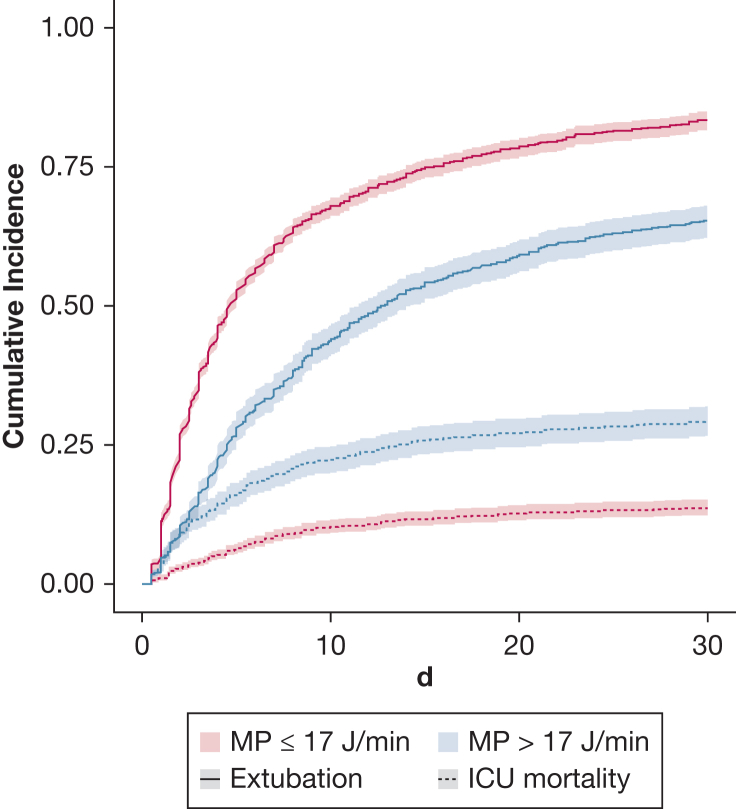
Graph showing the cumulative incidence curve up to day 30 (Fine and Gray subdistribution hazard model). High MP was associated with a decreased cumulative incidence of extubation and an increased cumulative incidence of ICU mortality. MP = mechanical power.

Of the 3,421 patients with complete data in our competing risk model, 2,685 patients were extubated successfully during their ICU stay. High MP was associated with a lower cause-specific hazard ratio of 0.68 (95% CI, 0.62-0.75) and subdistribution hazard ratio of 0.66 (95% CI, 0.60-0.73) for extubation, as well as a higher cause-specific hazard ratio of 1.21 (95% CI, 1.02-1.44) and subdistribution hazard ratio of 1.52 (95% CI, 1.29-1.81) for ICU mortality, up to 30 days (Fig 4, e-Table 5). Sixty-six percent of patients were extubated successfully beyond 30 days (e-Table 6). High MP was associated with fewer VFDs (–2.23 days; 95% CI, –2.79 to –1.66) when holding all other variables constant (e-Fig 6). High MP was associated with an increase in the nonpulmonary SOFA score at 48 hours, but not with any change in the norepinephrine equivalent at 48 hours or in the number of chest tube placements by day 7 (e-Tables 7-9).

### Subgroup and Sensitivity Analyses

The relationship between baseline MP and ICU mortality in the nonimputed dataset mirrors that of the imputed dataset, with comparable strength of association (Fig 2, e-Figs 3-5). Our decile analysis identified a cutoff point of 18.4 J/min, beyond which the association between MP and the odds of ICU mortality remained > 1 (e-Fig 7, e-Table 10). Severity of lung injury did not modify the association between high MP and ICU mortality (e-Figs 8, 9). The E-value for our PS model was 1.83 on the risk ratio scale (lower limit of the 95% CI at 1.69) (e-Fig 10). High MP, calculated using ΔP, was associated with a significantly increased ICU mortality (OR, 1.31; 95% CI, 1.12-1.50) (e-Figs 11-14).

## Discussion

In this multicenter registry-based cohort study of 9,031 adult patients with AHRF, exposure to high MP within the first 24 hours of IMV was associated significantly with higher ICU mortality. We observed a nonlinear association between MP and ICU mortality. Our threshold identification methods yielded different results, underscoring the challenge of defining a universal MP cutoff. Collectively, these findings suggest that MP operates as a continuous risk factor, with higher values associated with worse outcomes, rather than a distinct safe threshold. High MP was associated with a lower rate of extubation within the first 30 days and fewer VFDs at day 28. High MP also was associated with an increase in the nonpulmonary SOFA score at 48 hours, but not with any change in the norepinephrine equivalent at 48 hours or in the number of chest tube placements by day 7.

Our findings are consistent with previous research identifying a positive association between MP and patient outcomes.^19^, ^20^, ^21^, ^22^, ^23^, ^24^^,^^28^, ^29^, ^30^, ^31^^,^^41^^,^^42^ MP serves as a comprehensive and clinically relevant variable that encompasses all factors thought to be associated with the development of VILI. It integrates traditional parameters, such as V_T_ and ΔP, alongside often discarded variables, such as P_peak_ and respiratory rate.^17^ This holistic approach enables MP to present a more complete picture of the combined effects of various ventilatory parameters on patient outcomes, rather than isolating the impact of individual components within the MP equation. Previous studies provide compelling evidence that MP contributes to VILI across diverse ventilator settings,^28^, ^29^, ^30^, ^31^ including intraoperative ventilation,^30^^,^^43^ highlighting its relevance beyond patients in the ICU and the risks of injurious IMV settings. By focusing on patients with AHRF, our study extends the applicability of MP beyond ARDS, suggesting that MP could be a critical factor in the management of patients with AHRF, regardless of the underlying condition.

Although numerous studies have found an association between MP and VILI in animal and human models, no definitive threshold for patient harm has been identified. Guerin et al^20^ reported higher mortality rates among patients with ARDS with an MP of > 12 J/min, whereas Serpa Neto et al^19^ found that an MP of > 17 J/min in the second 24 hours of ventilation was associated with longer hospital length of stay and higher mortality. Parhar et al^21^ observed increased 28-day and 3-year mortality in patients with an MP of > 22 J/min at admission. However, recent pig studies challenge the concept of a clinically meaningful MP threshold, demonstrating VILI risk even at lower levels.^44^^,^^45^ Our findings align with these results, demonstrating an increase in ICU mortality risk at even lower MP levels. To our knowledge, our study is the first to explore the existence of an MP threshold in patients with AHRF, revealing that mortality increases even at lower MP levels, challenging the concept of a safe threshold. This insight is pivotal because it highlights the need for future research to focus on strategies aimed at achieving the lowest MP that is attainable safely, rather than relying on a specific threshold.

Currently, no established bedside protocol for reducing MP exists. Minimizing V_T_ and optimizing inspiration to expiration ratio effectively reduces P_peak_ and ΔP_dyn_, thereby lowering MP. Additionally, lung recruitment strategies through PEEP optimization and prone positioning improve compliance, further decreasing MP. The incorporation of PEEP into MP is complex and debated, because PEEP may be a marker of disease severity. Current models assume a linear relationship of PEEP with MP, although evidence suggests a U-shaped relationship between PEEP and VILI, influencing compliance and subsequent MP.^46^ Additional challenges of using MP at the bedside include the routine lack of static measurements, particularly in patients making spontaneous breathing efforts. To overcome this limitation, the use of ΔP_dyn_ in the calculation of MP has been proposed as a substitute for VILI risk assessment, applicable across all ventilation methods.^28^, ^29^, ^30^, ^31^ Easily calculated at the bedside, ΔP_dyn_ allows continuous monitoring of physiologic changes, facilitating patient-centered care through informed ventilation strategies and real-time feedback. Despite its lesser precision in estimating the total distending pressure of the respiratory system compared with ΔP, ΔP_dyn_ offers comparable information.^47^

Our study has several strengths. First, the Toronto Intensive Care Observational Registry is a large and comprehensive registry encompassing data from a diversity of patients at 7 hospitals. By including all patients with AHRF, regardless of their diagnosis, our results are generalizable to most patients receiving IMV, bypassing the challenges associated with the recognition of ARDS.^48^^,^^49^ Additionally, our results are independent of ventilatory methods, broadening the relevance of these findings beyond considerations of specific diagnoses or respiratory conditions. Although the use of a heterogeneous cohort may introduce variability, it also strengthens the robustness of our findings by reflecting real-world clinical practice in patients with AHRF. By examining the impact of MP, calculated using ΔP_dyn_, our study addresses an important gap in the literature. The incorporation of multiple sensitivity analyses further adds to the robustness and reliability of our results.

Our study has limitations. The PS analysis relies on the assumption of exchangeability, assuming the absence of unmeasured confounding. We addressed confounding during the design, and consistent results across sensitivity analyses enhance our confidence in the observed relationship. The E-value of 1.83 for our PS model indicates that an unmeasured confounder would need to have a risk ratio of at least 1.83 with both high MP and ICU mortality to nullify the observed association, assuming that all other measured confounders remain constant. To provide context, each 1-unit increase in the SOFA score corresponds to an OR of 1.10. Therefore, any unknown confounder influencing our observed association would have to be more influential than the severity of illness. The nonlinearity observed in the association between MP and ICU mortality is influenced by a limited number of patients, potentially impacting the true nature of the association. A significant number of patients did not meet our inclusion criteria at the time of initial data collection, affecting our sample size. However, this approach excluded patients with rapidly improving conditions for whom IMV settings may impart less risk.^2^^,^^50^ Respiratory parameters were recorded once daily (8 am), potentially missing intraday ventilation variations. For one-third of patients, altered level of consciousness was the reason for IMV; however, the cause of encephalopathy was not recorded, limiting further characterization. The cohort included patients receiving pressure support ventilation, a limitation because the MP equation assumes no spontaneous breathing. Although reflecting real-world practice, we addressed this in a sensitivity analysis restricted to controlled ventilation, confirming the robustness of our findings. To validate our findings and to discern potential disparities, future research should compare MP values derived from the equation with those obtained through pressure-volume loop analysis in spontaneously breathing patients. Cause of death, including withdrawal of life-sustaining therapies, was not recorded, preventing assessment of its variation with MP. Finally, although our data set contains a substantial amount of missing values, the consistency of results before and after multiple imputation supports the robustness of our findings.

## Interpretation

Exposure to an MP of > 17 J/min during the initial 24 hours of IMV, calculated using ΔP_dyn_, was associated independently with higher ICU mortality. We observed a nonlinear association between MP and ICU mortality, with increased risk of ICU mortality even at lower MP levels. Despite using various data-driven methods, no consistent safe MP threshold was identified. High MP was associated with a lower rate of extubation and fewer VFDs. These findings suggest that clinicians should consider limiting MP early in IMV to mitigate the risk of VILI. Prospective studies are needed to determine whether MP reduction improves patient outcomes.

## Funding/Support

S. v. D. is supported by a research grant from the Valeria Rossi di Montelera Foundation and by a Geneva University Hospitals grant for advanced training abroad. M. U. is supported by a scholarship from the Interdepartmental Division of Critical Care Medicine, 10.13039/501100003579University of Toronto, Toronto, ON, Canada.

## Financial/Nonfinancial Disclosures

The authors have reported to *CHEST* the following: S. v. D. received speaking honoraria from Löwenstein Medical. J. K. is a member of the data monitoring committee for a clinical trial sponsored by Eledon Pharmaceuticals, testing a novel therapeutic in kidney transplantation. E. F. reports personal fees from ALung Technologies, Baxter, Getinge, Inspira, Vasomune, and Zoll Medical outside the submitted work. None declared (K. L., L. M., M. U., N. K. J. A., K. K. S. P.).

## Supplementary Data

e-Online Data

Audio
