# Supplementary material for: The Association Between Mechanical Power Within the First 24 Hours and ICU Mortality in Mechanically Ventilated Adult Patients With Acute Hypoxemic Respiratory Failure: A Registry-Based Cohort Study
Source: Chest. 2025 Mar 28;168(4):901–11. doi: 10.1016/j.chest.2025.03.012 (PMC12597590; doi:10.1016/j.chest.2025.03.012)
Supplement: e-Online Data [file mmc1.pdf]

The association between mechanical power within the first 24 hours and ICU mortality in mechanically ventilated adult patients with acute hypoxemic respiratory failure: A registry-based cohort study

Online Data Supplement

CHEST
2025

Stephan von Düring, MD MSc
Kuan Liu, PhD
Laveena Munshi, MD MSc
S. Joseph Kim, MD PhD
Martin Urner, MD PhD
Neill KJ Adhikari, MDCM MSc
Ken Kuljit S. Parhar, MD MSc
Eddy Fan, MD PhD

Correspondence to:
Eddy Fan, MD, PhD
Interdepartmental Division of Critical Care Medicine (IDCCM),
University of Toronto, Toronto, ON, Canada

ONLINE DATA SUPPLEMENT

METHODS 4

Data source and study population 4

Mechanical power calculation..... 4

Propensity Score and Inverse Probability of Treatment Weight Analysis 5

e-Figure 1. Absolute standardized mean differences (SMD) between unweighted and weighted samples..... 6

Directed acyclic graph 7

e-Figure 2. Directed acyclic graph modelling exposure-outcome relationship 7

RESULTS 8

Missing data 8

e-Table 1. Description of missing data at baseline..... 8

e-Table 2. Standardized mean differences (SMD) between non-imputed and imputed datasets for baseline characteristics 10

Restricted cubic spline model selection 11

e-Table 3. Model fit evaluation the association between baseline MP and ICU mortality 11

e-Figure 3. Association between baseline mechanical power and ICU mortality in the complete case dataset..... 12

e-Figure 4. Association between baseline mechanical power and ICU mortality using the first imputed copy..... 12

e-Figure 5. Association between baseline mechanical power and ICU mortality by imputed copies 13

Change-point analyses 14

e-Table 4. Change point analysis between mechanical power and ICU mortality using the *chngptm* package 14

Competing risk 15

e-Table 5. Hazard ratios (95% confidence intervals) from Cause-Specific and Subdistribution Hazard models assessing the association between baseline mechanical power > 17 J/min and ICU mortality, and extubation, at 30 days..... 15

e-Table 6. Cumulative Incidence Function (CIF) estimates (95% confidence intervals) at day 7..... 15

and day 30 for ICU mortality and extubation, stratified by baseline mechanical power at 17 J/min 15

Ventilator-free days..... 16

e-Figure 6. Distribution of ventilator-free days by baseline mechanical power at 17 J/min for survivors..... 16

Disease severity and chest tubes placement..... 17

e-Table 7. Adjusted linear regression between baseline mechanical power and non-pulmonary SOFA score at 48 hours 17

e-Table 8. Adjusted linear regression between baseline mechanical power and norepinephrine equivalent at 48 hours 17

e-Table 9. Adjusted negative binomial regression between baseline mechanical power and chest tubes at 17

day 7 17

Decile analysis 18

e-Figure 7. Association between baseline mechanical power deciles and ICU mortality 18

e-Table 10. Association between baseline mechanical power (10% deciles) and ICU mortality..... 18

Subgroup Analysis 19
e-Figure 8. Association between baseline mechanical power > 17 J/min and ICU mortality, stratified by severity of lung injury. 19
e-Figure 9. Association between baseline mechanical power > 17 J/min and ICU mortality, stratified by severity of lung injury. 20
e-Figure 10. Bias plot 21
Mechanical Power, calculated using static ΔP (P_{plat} – PEEP) 22
e-Figure 11. Association between mechanical power at baseline and ICU mortality using complete case analysis 22
e-Figure 12. Association between mechanical power at baseline and ICU mortality using the first imputed copy23
e-Figure 13. Association between mechanical power at baseline and ICU mortality by imputed copies 24
e-Figure 14. Association between mechanical power above 17 J/min at baseline and ICU morality 25
REFERENCES 26

METHODS

Data source and study population

The Toronto Intensive Care Observational Registry (iCORE) comprises prospectively collected data from adult patients who received mechanical ventilation for ≥ 4 hours in nine ICUs affiliated with the University of Toronto. Our study used data collected from April 11, 2014 (inception) until May 31, 2023. Data collection occurred once daily at 8 AM, using a hybrid approach, combining automated data extraction from electronic patient records (EPR) for clinical variables, blood gas, and laboratory measurements, and manual collection of demographic characteristics, ventilation parameters, and patient outcomes.

In our study, patients who received ventilation via tracheostomy were included, and their data were not distinguished in the analysis. In cases where patients had multiple ICU admissions within the same hospital stay, we utilized data from their initial admission for our analysis.

Mechanical power calculation

Our primary exposure, MP, was calculated once for each patient within the first 24 hours of IMV, representing the initial lung parameters closest to the time of initiation of IMV.

Mechanical power (MP) was calculated using the following equation:

$$MP\ (J/min) = RR \times V_T \times (P_{peak} - (\Delta P)/2) \times 0.098$$ [1]

MP: mechanical power
RR: respiratory rate
V_T: tidal volume
P_{peak}: peak pressure
ΔP: driving pressure

ΔP is defined as the inspiratory pressure - PEEP.

MP using static ΔP (ΔP) = P_{plat} – PEEP[2]

MP using dynamic ΔP (ΔP_{dyn}) = P_{peak} - PEEP[3-6]

PEEP: positive end expiratory pressure
P_{plat}: plateau pressure

Propensity Score and Inverse Probability of Treatment Weight Analysis

The propensity score model included variables identified as confounders based on our DAG (e-Figure 2) and variables known to be solely associated with the outcome, ensuring that associations were grounded in clinical intuition rather than data-driven variable selection procedures.[7] To maintain data integrity, we assessed the similarity of variable distributions before and after imputation (e-Table 10).

In line with a trial-like approach and guided by a conceptual threshold identified in a previous work[8], we classified the intervention as high MP (> 17 J/min) versus low MP (≤ 17 J/min). The propensity score was estimated using a logistic regression model, regressing treatment assignment on the following selected variables (age, sex, body mass index (BMI), APACHE III score, SOFA score, mean arterial pressure (MAP), lactate, comorbidities at baseline (cardiovascular, diabetes, respiratory, active neoplasm, chronic kidney disease, immunosupression and AIDS, liver failure), reason for mechanical ventilation (acute respiratory failure, altered level of consciousness, acute exacerbation of chronic pulmonary disease, neuromuscular causes, not available), tidal volume per predicted body weight (PBW) after intubation, PaO_2/FiO_2 after intubation, arterial pH after intubation, ventilatory ratio). Each patient was then weighted using the inverse probability of treatment weighting (IPTW) to account for observed confounding imbalances, with stabilization and trimming at the 1st and 99th percentiles applied to address extreme weights.

Standardized mean differences (SMD) were calculated to assess the balance in measured baseline covariates between groups.[9] After modeling the relationship between ventilation ratio and the log-odds of MP using a restricted cubic spline with four knots, appropriate balancing of baseline covariates was achieved. Standardized differences of more than 0.2 were considered an indicator of covariates imbalance after PS weighting.[10] Apart from ventilation ratio, which had a standardized mean difference of 0.15, all other variables in the model had a standardized mean difference < 10%, indicating good balance between the selected covariates in the two groups (e-Figure 1).

e-Figure 1. Absolute standardized mean differences (SMD) between unweighted and weighted samples

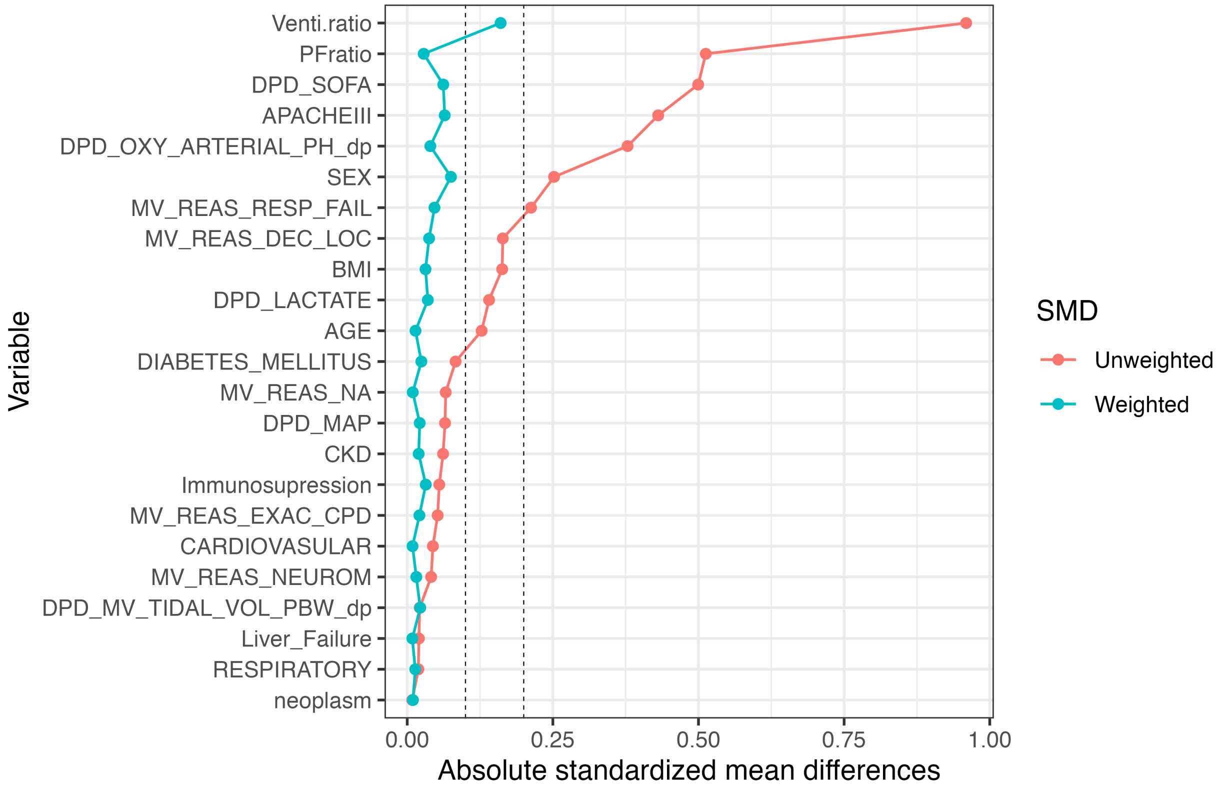

The dotted vertical lines represent SMD thresholds of 0.1 and 0.2.
SMD, standardized mean difference

Directed acyclic graph

A directed acyclic graph (DAG) was constructed to illustrate the hypothesized relationships between the primary exposure, confounding variables, and primary outcome variable. Based on prior data and clinical expertise, several potential confounders were identified in the association between MP and intensive care unit (ICU) mortality. These confounders include sex, age, body mass index (BMI), severity-of-disease (APACHE III and SOFA score), severity of hypoxemia (PaO_2/FiO_2 ratio), and pH.

1. Women have been observed to exhibit higher illness severity scores at ICU admission and a higher mortality rate compared to men.[11,12]
2. Age has been associated with increased compliance[13] and a direct and indirect increase in ICU mortality.[14,15]
3. A higher BMI has been linked to an elevated risk of mortality in the ICU.[16,17]
4. The APACHE III score, which incorporates age, PaO_2/FiO_2 ratio, and pH, influences both MP and ICU mortality.[18]
5. Similarly, the SOFA score, which incorporates the PaO_2/FiO_2 ratio, affects MP and ICU mortality.[18]
6. The PaO_2/FiO_2 ratio itself can modify MP through positive end-expiratory pressure (PEEP) and independently impact ICU mortality.[19]
7. Lastly, pH, which can modify MP through minute ventilation, is associated with both APACHE III score and ICU mortality.[20,21]

e-Figure 2. Directed acyclic graph modelling exposure-outcome relationship

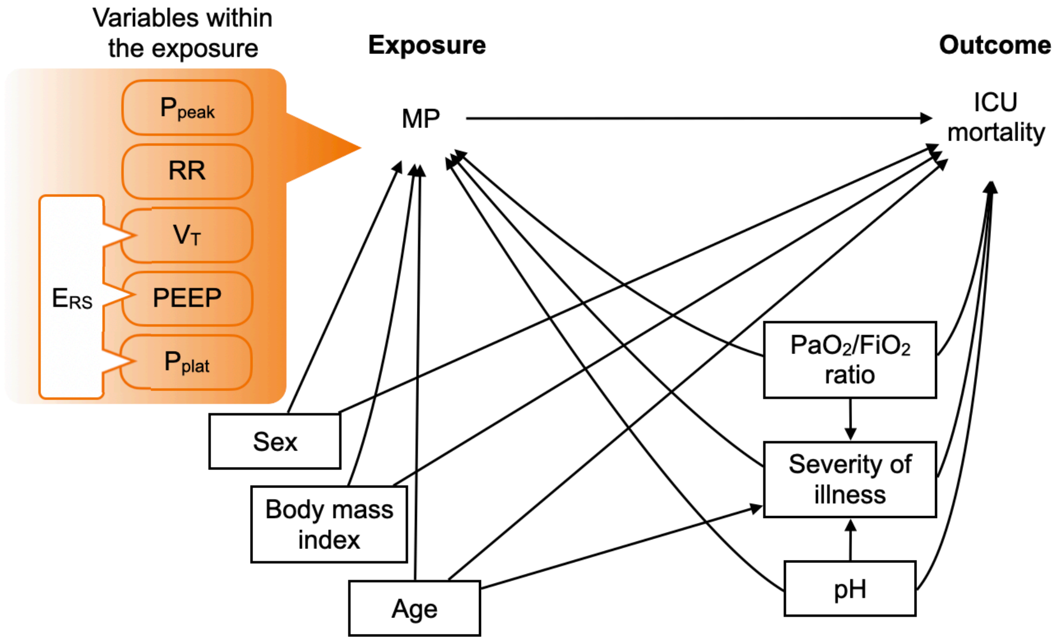

To address these confounding factors, multivariable regression models were employed to adjust for them during the analysis. The same causal diagram was used to estimate additional study endpoints to ensure consistency in the modeling approach. *E_{RS}, elastance of the respiratory system; P_{peak}, peak pressure; RR, respiratory rate; V_T, tidal volume; PEEP, positive end-expiratory pressure; P_{plat}, plateau pressure; MP, mechanical power; ICU, intensive care unit. Severity of illness encompasses the following scores: APACHE, Acute Physiology and Chronic Health Evaluation III; SOFA, Sequential Organ Failure Assessment.*

RESULTS

Missing data

Static ventilatory parameters, including plateau pressures, static ΔP, and MP, are typically measured for patients on controlled modes of ventilation, which are primarily utilized for a minority of patients with critical respiratory support requirements. Consequently, in larger ICU patient populations, these static measurements are often not routinely recorded, as demonstrated by the LungSafe study, which reported plateau pressure and driving pressure measurements for only 40.1% of all patients.[22]

e-Table 1. Description of missing data at baseline

Variable	Missing values (n)	Missing values (%)
Static Elastance normalized to PBW	8183	90.6
Mechanical power, using ΔP	7960	88.1
Plateau pressure (P_{plat})	7938	87.9
Driving pressure (ΔP)	7938	87.9
Norepinephrine equivalents	5657	62.6
Lactate	5638	62.4
Mean arterial pressure	4745	52.5
Ventilatory ratio	4530	50.2
Body mass index	4322	47.9
Tidal volume by PBW	3069	34.0
Height	3024	33.5
Weight	2574	28.5
PaO_2	2332	25.8
PaO_2/FiO_2 ratio	2332	25.8
Arterial pH after intubation	2330	25.8
$PaCO_2$ after intubation	2328	25.8
Mechanical power, using ΔP_{dyn}	508	5.6
Peak inspiratory pressure (P_{peak})	448	5.0
Dynamic driving pressure(ΔP_{dyn})	448	5.0
APACHE III score	295	3.3
Tidal volume	65	0.7
SpO_2	32	0.4
SpO_2/FiO_2 ratio	32	0.4
Observed respiratory rate	12	0.1
Age	0	0.0
Biological sex	0	0.0
SOFA score	0	0.0
FiO_2	0	0.0
Positive end-expiratory pressure (PEEP)	0	0.0
Length of mechanical ventilation	0	0.0
ICU Length of stay	0	0.0
Discharge disposition	0	0.0

PBW, predicted body weight; PaO_2, partial pressure of oxygen in arterial blood; PaO2/FiO2, ratio of partial pressure of oxygen to fraction of inspired oxygen concentration in arterial blood; $PaCO_2$, partial pressure of carbon dioxide in arterial blood; SpO_2, percentage of oxygen in the blood; PpO_2/FiO_2, ratio of percentage of oxygen to fraction of inspired oxygen concentration; FiO_2, fraction of inspired oxygen; ICU, intensive care unit.

To address uncertainty regarding missing data at baseline, we employed multiple imputation by chained equations (*MICE* package[23]) and generated 5 imputed datasets for the full study. We examined missing data patterns and assessed data distribution before and after imputation using summary statistics (e-Table 11). Our predictor matrix was composed of 95 variables, including baseline characteristics, comorbidities, ventilator settings, blood gas measurements, and outcome. We used the built-in univariate imputation methods to determine values for our missing data (logistic regression (logreg) for binary variables, polytomous regression (polyreg) for categorical variable, proportional odds model (polr) for multiple ordered levels, and predictive mean matching (pmm) for numeric variables). Imputed data were used in the primary analysis on ICU mortality, with model output pooled using Rubin's rules.[24] MP is a composite variable that depends on RR, V_T, P_{peak} and PEEP, and was calculated pre and post-MICE procedure after imputing missing values for these variables.

e-Table 2. Standardized mean differences (SMD) between non-imputed and imputed datasets for baseline characteristics

	Complete case data	Imputed data
	SMD	SMD
**Baseline characteristics**		
Age, years	0.14	0.13
Female	0.26	0.25
Weight, Kg	0.38	0.25
APACHE III score, points	0.45	0.43
SOFA score, points	0.51	0.50
**Comorbidities**		
Cardiovascular	0.05	0.04
Diabetes	0.08	0.08
Respiratory	0.02	0.02
Active neoplasm	0.01	0.01
Chronic kidney disease	0.07	0.06
Immunosupression and AIDS	0.05	0.06
Liver failure	0.02	0.02
**Reason for intubation**		
Acute respiratory failure	0.21	0.21
Altered level of consciousness	0.16	0.16
Acute exacerbation of chronic pulmonary disease	0.05	0.05
Neuromuscular causes	0.04	0.04
Not available	0.07	0.07
**Blood gasses exchange and ventilator parameters**		
PaO_2/FiO_2 after intubation, mmHg	0.59	0.51
SpO_2/FiO_2 after intubation	0.85	0.82
$PaCO_2$ after intubation, mmHg	0.12	0.09
Arterial pH after intubation	0.49	0.38
Tidal volume by PBW, mL/Kg	0.03	0.09
MP, J/min	2.5	2.5

Baseline data was collected at 8 AM within the first 24 hours of invasive mechanical ventilation.

SMD, standardized mean difference; APACHE: acute physiology and chronic health evaluation; SOFA, sequential organ failure assessment;

PaO_2, partial pressure of oxygen; FiO_2, fraction of inspired oxygen; SpO_2, percentage of oxygen in the blood; $PaCO_2$, partial pressure of bicarbonate; MP, mechanical power; J/min, Joules per minute.

Restricted cubic spline model selection

To evaluate the relationship between MP and ICU mortality in our explanatory model, while considering non-linear associations, we employed spline modeling. We aimed to strike a balance between model flexibility and overfitting, so we manually determined the optimal number of knots for each variable in our regression model.[25] Using the likelihood-ratio test, we found that the 3435343_model, which included 3 knots for MP, 4 knots for age, 3 knots for BMI, 5 knots for APACHE III, 3 knots for SOFA, 4 knots for PaO_2/FiO_2 ratio, and 3 knots for pH, demonstrated the best fit among the tested models, on both complete case and imputed datasets ($P < 0.0001$).

e-Table 3. Model fit evaluation the association between baseline MP and ICU mortality

	Complete case analysis		Imputed dataset	
Model	Linear model	Restricted cubic spline	Linear model	Restricted cubic spline
AIC	3100	3059	8506	8401
C-index	0.74	0.77	0.75	0.76

Complete case dataset, N after adjusting = 3,421. Imputed dataset, m = 5, N = 9,031.
AIC, Akaike information criterion; C-index, concordance statistic.

e-Figure 3. Association between baseline mechanical power and ICU mortality in the complete case dataset

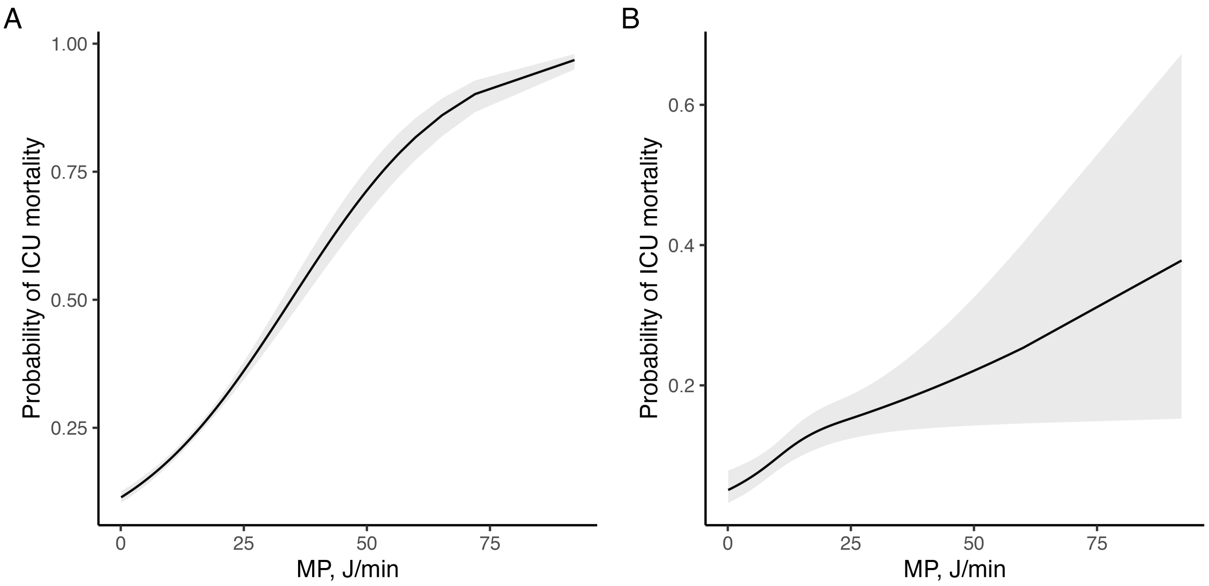

Mean predicted effect of MP on the probability of ICU mortality. (A) Unadjusted linear regression model with only the intercept as a predictor variable (N = 8523). (B) Multivariate restricted cubic spline regression model, adjusting for the confounders identified in our directed acyclic graph (N = 3421).
ICU, intensive care unit; MP, mechanical power; J/min, Joules per minute.

e-Figure 4. Association between baseline mechanical power and ICU mortality using the first imputed copy

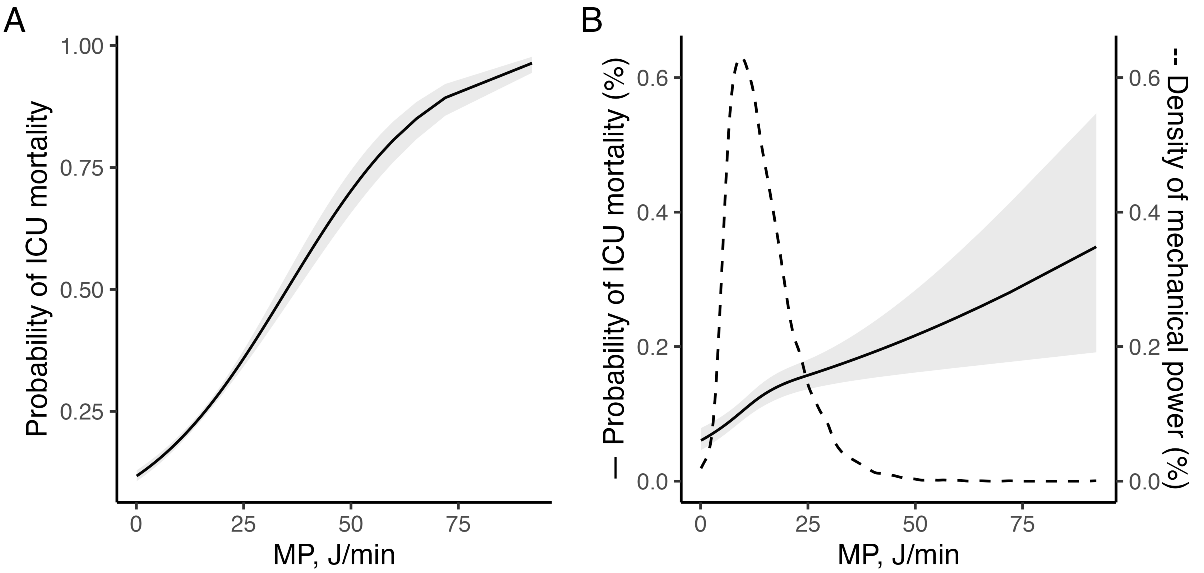

Mean marginal predicted effect of MP on ICU mortality. This was computed from a restricted cubic spline regression model using the first copy of the imputed dataset (N = 9031), and adjusting for confounders in our DAG. (A) Unadjusted linear regression model with only the intercept as a predictor variable. (B) Multivariate restricted cubic spline regression model, adjusting for the confounders identified in our directed acyclic graph.
Copies 2-3-4-5 of the imputed dataset are presented as e-Figure 5.
ICU, intensive care unit; MP, mechanical power; J/min, Joules per minute.

e-Figure 5. Association between baseline mechanical power and ICU mortality by imputed copies

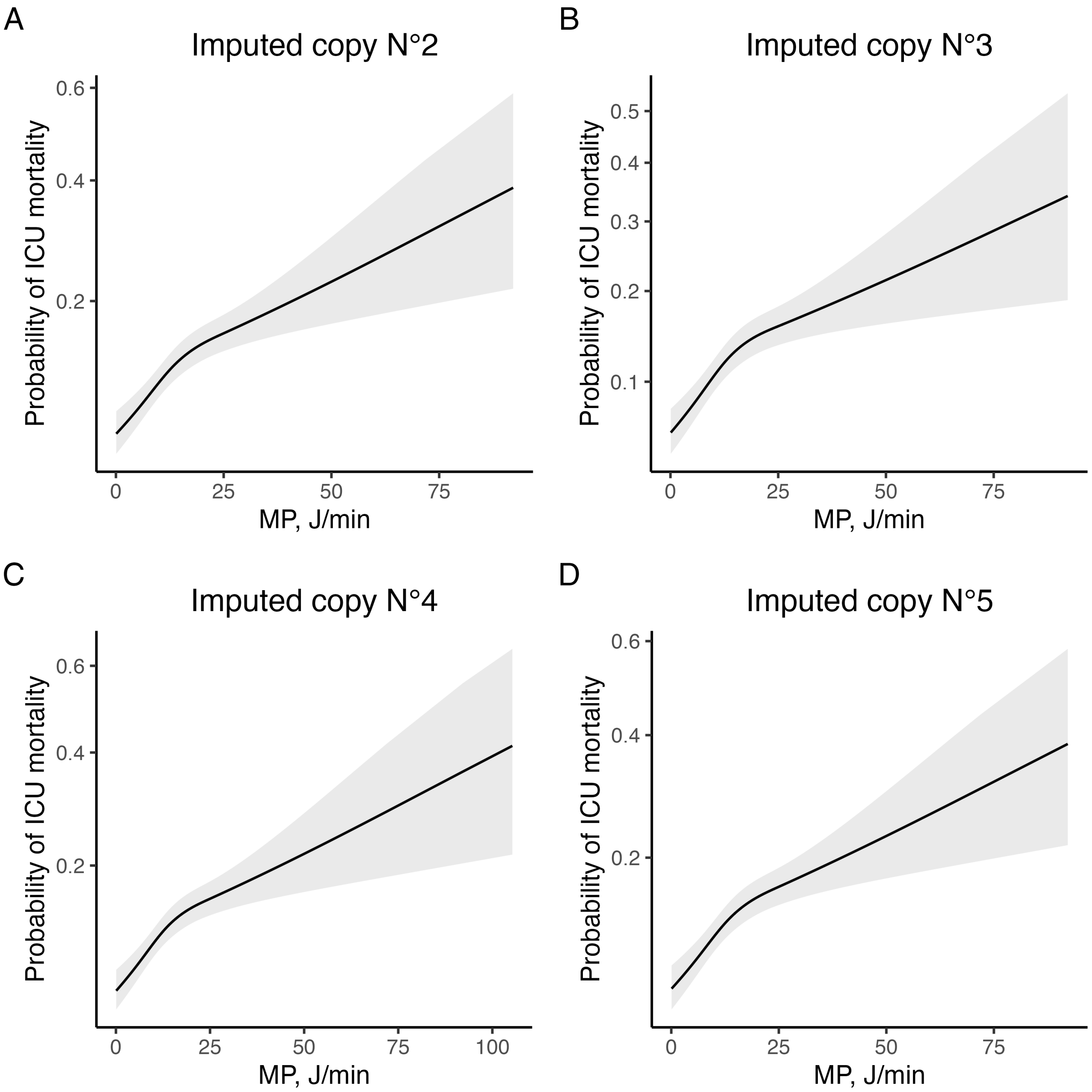

Mean marginal predicted effect of MP on ICU mortality. This was computed from a restricted cubic spline regression model using copies 2-3-4-5 of the imputed dataset (N = 9031), and adjusting for confounders in our DAG. Copy 1 of the imputed dataset is presented as e-Figure 4.

ICU, intensive care unit; MP, mechanical power; J/min, Joules per minute.

Change-point analyses

e-Table 4. Change point analysis between mechanical power and ICU mortality using the *chngptm* package

**Complete case analysis**	**Change point**	**95% CI**
multivariate LR	27.86	6.27, 30.96
spline model	28.04	6.27, 30.96
**Imputed data**		
multivariate LR	7.11	6.17, 29.01
spline model	7.21	6.20, 29.35

LR, logistic regression; CI, confidence interval

Competing risk

e-Table 5. Hazard ratios (95% confidence intervals) from Cause-Specific and Subdistribution Hazard models assessing the association between baseline mechanical power > 17 J/min and ICU mortality, and extubation, at 30 days.

	Cause-Specific Hazard Model		Subdistribution Hazard Model	
**Vaiables**	**ICU mortality**	**Extubation**	**ICU mortality**	**Extubation**
MP >17	1.21 (1.02, 1.44)	0.68 (0.62, 0.75)	1.52 (1.29, 1.81)	0.66 (0.60, 0.73)
Age	1.00 (1.00, 1.01)	1.00 (1.00, 1.01)	1.00 (0.99, 1.01)	1.00 (0.99, 1.00)
Female	1.03 (0.88, 1.21)	0.93 (0.85, 1.00)	1.06 (0.90, 1.25)	0.91 (0.84, 0.99)
BMI	0.99 (0.98, 1.00)	1.00 (1.00, 1.01)	0.99 (0.98, 0.99)	1.00 (0.99, 1.01)
APACHE III score	1.01 (1.01, 1.02)	1.00 (0.99, 1.00)	1.02 (1.01, 1.02)	0.99 (0.99, 0.99)
SOFA score	1.05 (1.02, 1.07)	0.97 (0.96, 0.99)	1.06 (1.03, 1.08)	0.96 (0.95, 0.97)
PaO_2/FiO_2 ratio	1.00 (0.99, 1.01)	1.01 (1.00, 1.01)	0.99 (0.98, 0.99)	1.00 (1.00, 1.01)
Arterial pH	0.97 (0.97, 0.98)	1.01 (1.00, 1.01)	0.97 (0.96, 0.98)	1.01 (1.01, 1.02)

MP, mechanical power; ICU, intensive care unit; BMI, body mass index; APACHE, Acute Physiology and Chronic Health Evaluation III; SOFA, Sequential Organ Failure Assessment.

e-Table 6. Cumulative Incidence Function (CIF) estimates (95% confidence intervals) at day 7 and day 30 for ICU mortality and extubation, stratified by baseline mechanical power at 17 J/min

	Day 7		Day 30		***P* value***
**ICU mortality**					
MP					<0.001
≤ 17 J/min	8.40%	(7.3%, 9.6%)	14%	(12%, 15%)	
> 17 J/min	20%	(17%, 22%)	29%	(27%, 32%)	
**Extubation**					
MP					<0.001
≤ 17 J/min	61%	(59%, 63%)	83%	(82%, 85%)	
> 17 J/min	35%	(32%, 38%)	65%	(63%, 68%)	

MP, mechanical power; J/min, Joules per minute. *Gray's Test

Ventilator-free days

Ventilator-free days (VFD) was measured in days from intubation to successful extubation. Successful extubation was defined as truly liberated from mechanical ventilation for ≥ 2 consecutive days. This was a composite outcome of length of IMV and mortality. It was calculated as 28 days minus the number of calendar days intubated. Only data from the last successful extubation was used. For patients who stayed longer than 28 days in the ICU, VFD was set to 0 for right censoring. To penalize patients that died within the first 28 days, VFD was set to -1. This is to differentiate them from patients that died after 28-day.

e-Figure 6. Distribution of ventilator-free days by baseline mechanical power at 17 J/min for survivors

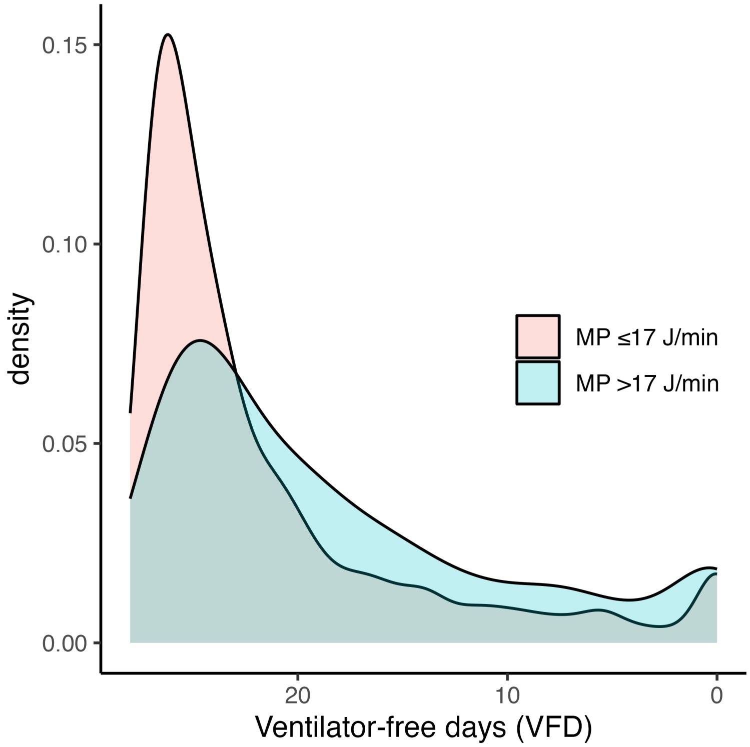

MP, mechanical power; J/min, Joules per minute.

Disease severity and chest tubes placement

e-Table 7. Adjusted linear regression between baseline mechanical power and non-pulmonary SOFA score at 48 hours

Variables	β coefficient	95% CI	*P* value
MP >17 J/min	0.40	0.23, 0.57	< 0.001
MP (continuous)	0.03	0.02, 0.04	< 0.001

Complete case analysis (N = 4540). Weighted regression model accounting for missing data due to early patient discharge (before 48 hours).
SOFA, sequential organ failure assessment; MP, mechanical power; J/min, Joules per minute.

e-Table 8. Adjusted linear regression between baseline mechanical power and norepinephrine equivalent at 48 hours

Variables	β coefficient	95% CI	*P* value
MP >17 J/min	11.2	-8.01, 30.35	0.30
MP (continuous)	0.78	-0.03, 1.58	0.11

Complete case analysis (N = 29). Weighted regression model accounting for missing data due to early patient discharge (before 48 hours).
SOFA, sequential organ failure assessment; MP, mechanical power; J/min, Joules per minute.

e-Table 9. Adjusted negative binomial regression between baseline mechanical power and chest tubes at day 7

Variables	β coefficient	95% CI	*P* value
MP >17 J/min	-0.01	-0.07, 0.06	0.88
MP (continuous)	-0.01	-0.01, 0.01	0.75

Complete case analysis (N = 1466). Weighted regression model accounting for missing data due to early patient discharge (before day 7).
SOFA, sequential organ failure assessment; MP, mechanical power; J/min, Joules per minute.

Decile analysis

By dividing our cohort into 10 groups (deciles) of MP, we aimed to identify the cutoff point at which the odds ratio of ICU mortality showed a statistically significant change compared to the overall median in the cohort.

e-Figure 7. Association between baseline mechanical power deciles and ICU mortality

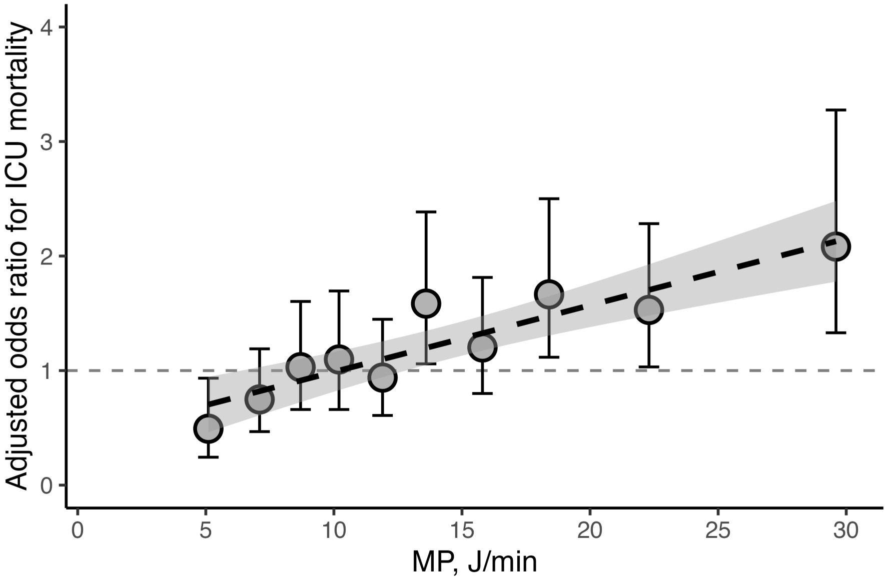

The complete case dataset (N = 8523) was stratified into 10 groups (deciles) of MP, with each group representing 10% of the cohort. Adjusted odds ratios (OR) for ICU mortality were calculated in relation to the median MP of the whole cohort (12.7 J/min). OR were adjusted for covariates in our DAG using a logistic regression model with a restricted cubic spline for MP. Error bars represent 95% confidence intervals around the OR estimates. We identified a cutoff point of 18.4 J/min, beyond which the association between MP and the odds of ICU mortality remained above 1. *ICU, intensive care unit; OR, odds ratio; MP, mechanical power; J/min, Joules per minute.*

e-Table 10. Association between baseline mechanical power (10% deciles) and ICU mortality

%	MP	OR	95% CI
5	5.1	0.49	0.24, 0.93
15	7.1	0.75	0.47, 1.19
25	8.7	1.03	0.66, 1.60
35	10.2	1.10	0.66, 1.70
45	11.9	0.94	0.61, 1.45
55	13.6	1.58	1.06, 2.39
65	15.8	1.20	0.80, 1.81
75	18.4	1.67	1.12, 2.50
85	22.3	1.53	1.03, 2.28
95	29.6	2.08	1.33, 3.28

ICU, intensive care unit; MP, mechanical power; OR, odds ratio; CI, confidence interval.

Subgroup Analysis

e-Figure 8. Association between baseline mechanical power > 17 J/min and ICU mortality, stratified by severity of lung injury.

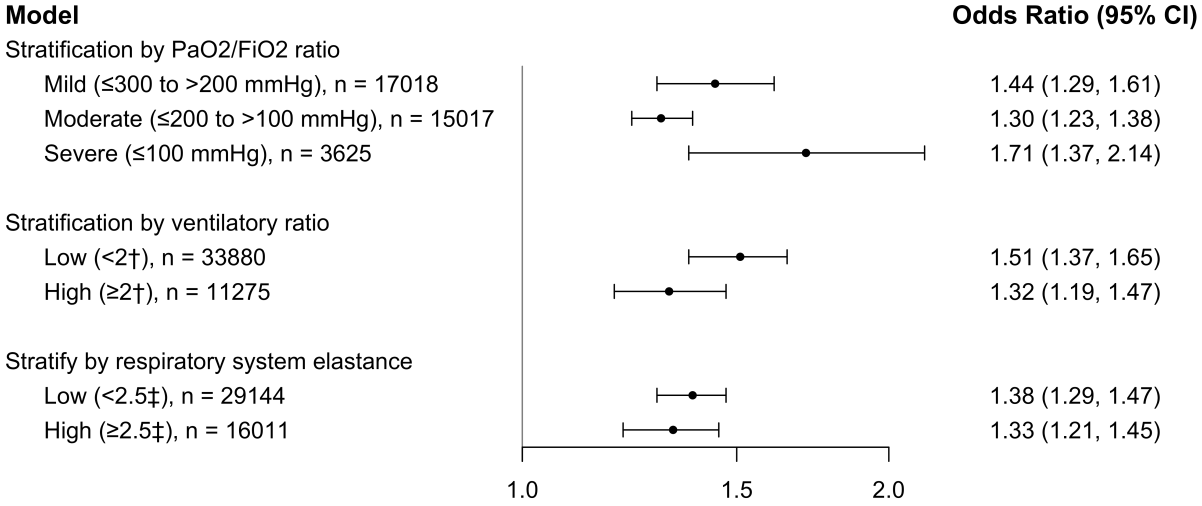

Analysis performed on the imputed datasets.
ICU, intensive care unit.
†(minute ventilation x $PaCO_2$)/(PBW × 100 × 37.5).
‡Calculation of respiratory system elastance was based on dynamic values, (cmH_2O/(mL/Kg PBW).

e-Figure 9. Association between baseline mechanical power > 17 J/min and ICU mortality, stratified by severity of lung injury.

Model	Odds Ratio (95% CI)	p-value*
Stratification by PaO2/FiO2 ratio		0.26
Mild (≤300 to >200 mmHg), n = 2580	1.53 (1.08, 2.16)	
Moderate (≤200 to >100 mmHg), n = 2186	1.32 (0.95, 1.84)	
Severe (≤100 mmHg), n = 595	1.80 (0.97, 3.35)	
Stratification by ventilatory ratio		0.95
Low (<2†), n = 3390	1.50 (1.16, 1.93)	
High (≥2†), n = 1111	1.49 (0.99, 2.27)	
Stratify by respiratory system elastance		0.26
Low (<2.5‡), n = 3637	1.24 (0.92, 1.67)	
High (≥2.5‡), n = 1974	1.43 (1.06, 1.92)	

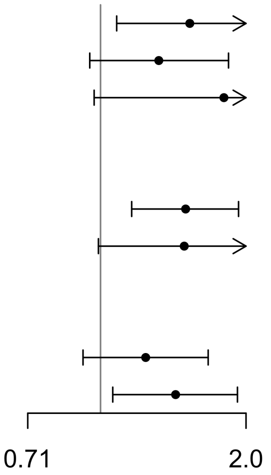

Analysis performed on the complete case dataset.
ICU, intensive care unit.
*Statistically significant *P* values threshold has been adjusted using the Bonferroni correction and is now defined as ≤ 0.007 to account for multiple testing and adjust the type 1 error.
†(minute ventilation x $PaCO_2$)/(PBW × 100 × 37.5).
‡Calculation of respiratory system elastance was based on dynamic values, (cmH_2O/(mL/Kg PBW).

E-value

e-Figure 10. Bias plot

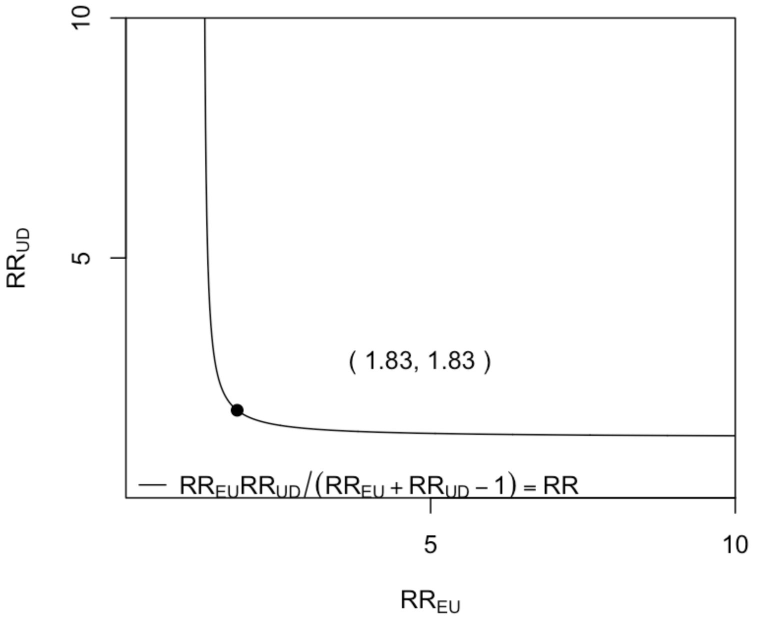

E-value was 1.83 (95% CI: 1.69, NA) for our propensity score model. For a reference, SOFA score had an OR of 1.10 (95% CI: 1.07, 1.12), for each 1-unit increment increase in the score. *RR_{EU}, exposure-confounder parameter; RR_{UD}, confounder-outcome parameter; RR, risk ratio; CI, confidence interval; OR, odds ratio.*

Mechanical Power, calculated using static ΔP (P_{plat} – PEEP)

e-Figure 11. Association between mechanical power at baseline and ICU mortality using complete case analysis

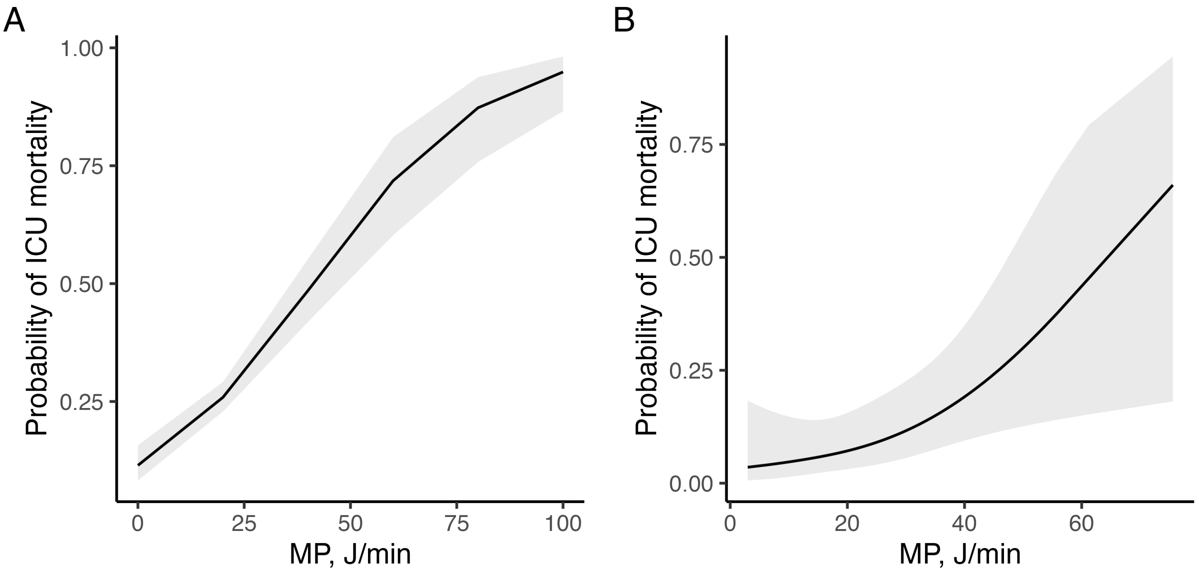

We calculated the mean predicted effect of mechanical power on the probability of ICU mortality. (A) Unadjusted linear regression model with only the intercept as a predictor variable, (N = 849). (B) Multivariate restricted cubic spline regression model, adjusting for the confounders identified in our directed acyclic graph, (N = 319). MP was calculated using static ΔP (P_{plat} – PEEP).

ICU, intensive care unit; MP, mechanical power; J/min, Joules per minute; P_{plat}, *plateau pressure; PEEP, positive end-expiratory pressure.*

e-Figure 12. Association between mechanical power at baseline and ICU mortality using the first imputed copy

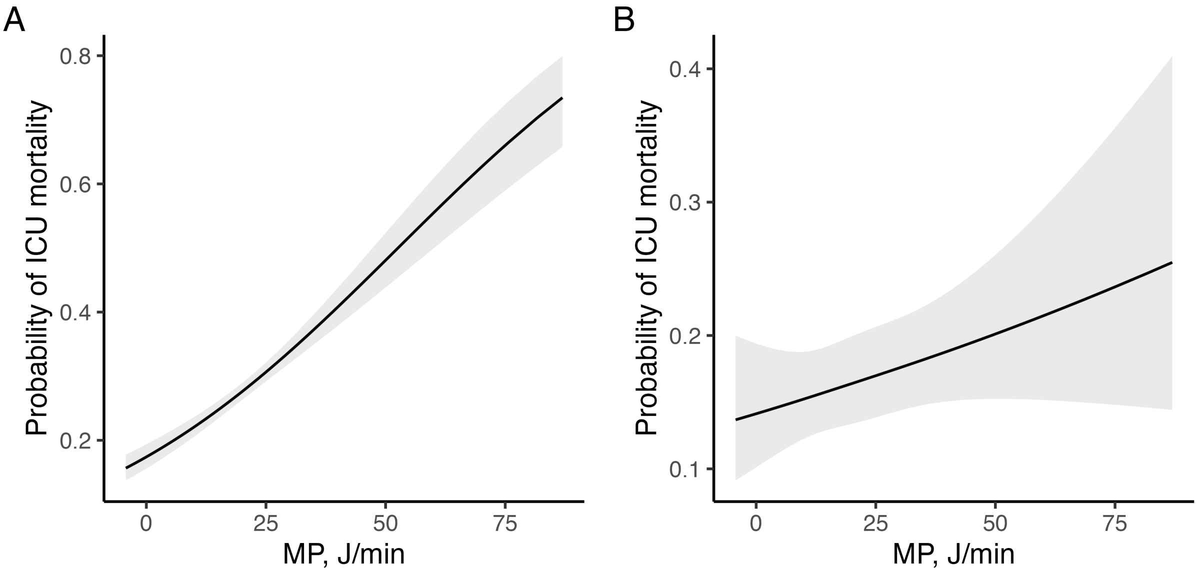

Mean predicted effect of statMP on the probability of ICU mortality. Figure **A** employed an unadjusted linear regression model with only the intercept as a predictor variable. Figure **B** employed a multivariate restricted cubic spline regression model, adjusting for the confounders identified in our directed acyclic graph (DAG), using the first copy of the imputed dataset (N = 4551).

ICU, intensive care unit; MP, mechanical power; J/min, Joules per minute.

e-Figure 13. Association between mechanical power at baseline and ICU mortality by imputed copies

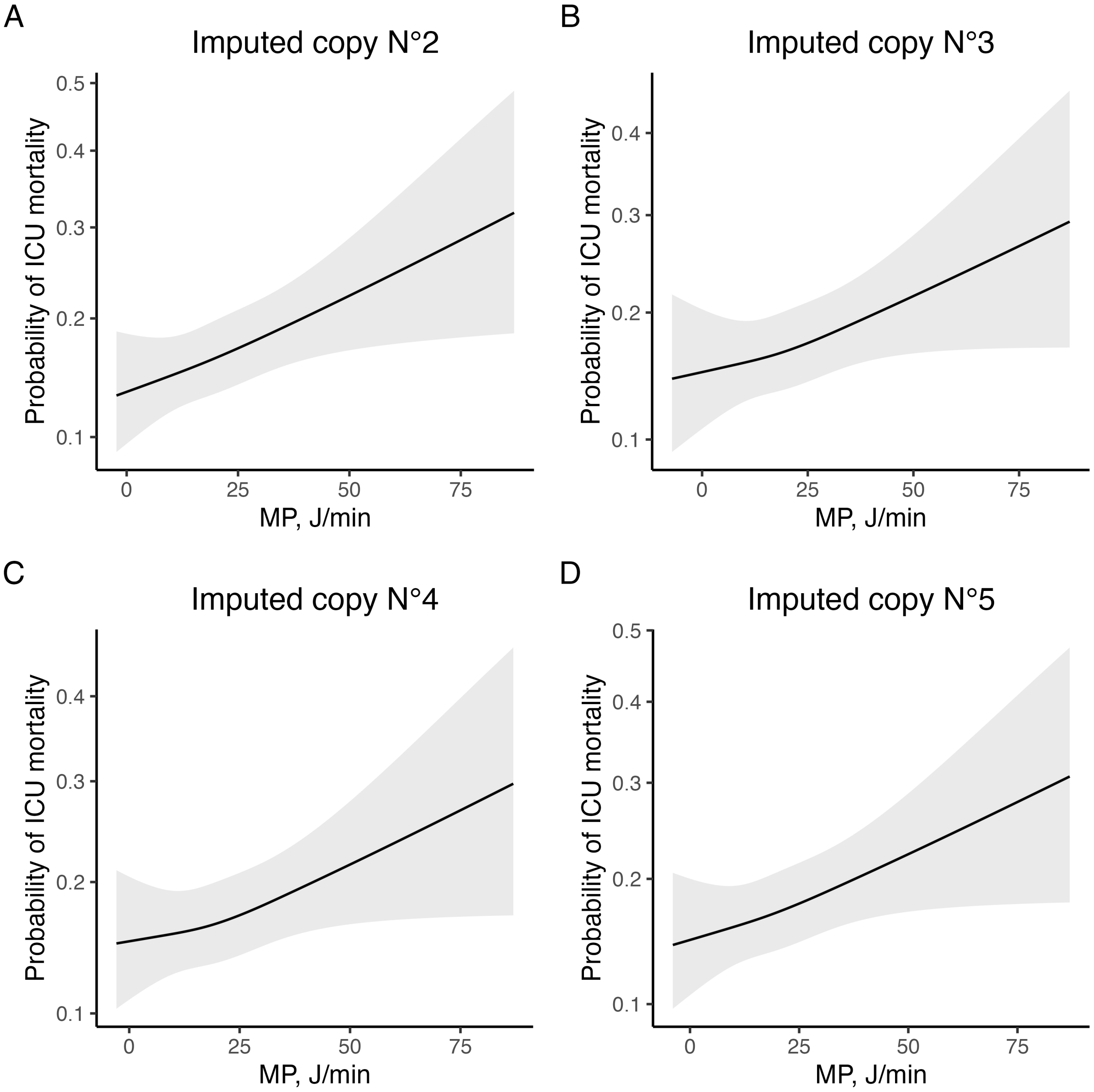

Mean marginal predicted effect of mechanical power on ICU mortality. This was computed from a restricted cubic spline regression model using copies 2-3-4-5 of the imputed dataset, and adjusting for confounders in our DAG (N = 4551).

ICU, intensive care unit; MP, mechanical power; J/min, Joules per minute.

e-Figure 14. Association between mechanical power above 17 J/min at baseline and ICU morality

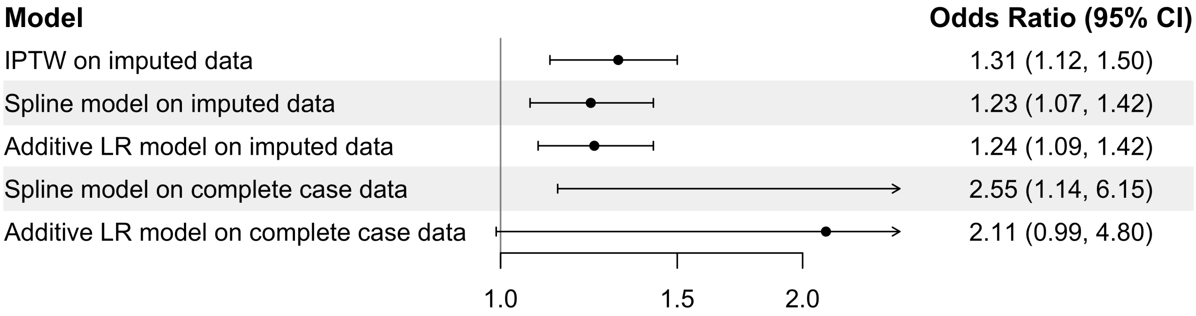

Complete case data, N after adjusting = 319. Imputed data, N = 5 x 4551.
CI, confidence interval; LR, logistic regression; IPTW, inverse probability of treatment weighting.

REFERENCES

1. Gattinoni L, Tonetti T, Cressoni M, et al. Ventilator-related causes of lung injury: the mechanical power. *Intensive care medicine.* 2016;42(10):1567-1575.
2. Amato MB, Meade MO, Slutsky AS, et al. Driving pressure and survival in the acute respiratory distress syndrome. *N Engl J Med.* 2015;372(8):747-755.
3. Urner M, Juni P, Hansen B, Wettstein MS, Ferguson ND, Fan E. Time-varying intensity of mechanical ventilation and mortality in patients with acute respiratory failure: a registry-based, prospective cohort study. *Lancet Respir Med.* 2020;8(9):905-913.
4. Schuijt MTU, van Meenen DMP, Martin-Loeches I, et al. Association of Time-Varying Intensity of Ventilation With Mortality in Patients With COVID-19 ARDS: Secondary Analysis of the PRoVENT-COVID Study. *Front Med (Lausanne).* 2021;8:725265.
5. Schuijt MTU, Hol L, Nijbroek SG, et al. Associations of dynamic driving pressure and mechanical power with postoperative pulmonary complications-posthoc analysis of two randomised clinical trials in open abdominal surgery. *EClinicalMedicine.* 2022;47:101397.
6. Chen H, Chen Z-Z, Gong S-R, Yu R-G. Visualizing the dynamic mechanical power and time burden of mechanical ventilation patients: an analysis of the MIMIC-IV database. *Journal of Intensive Care.* 2023;11(1):58.
7. Hernán MA, Hernández-Díaz S, Werler MM, Mitchell AA. Causal knowledge as a prerequisite for confounding evaluation: an application to birth defects epidemiology. *Am J Epidemiol.* 2002;155(2):176-184.
8. Serpa Neto A, Deliberato RO, Johnson AEW, et al. Mechanical power of ventilation is associated with mortality in critically ill patients: an analysis of patients in two observational cohorts. *Intensive care medicine.* 2018;44(11):1914-1922.
9. Austin PC, Stuart EA. Moving towards best practice when using inverse probability of treatment weighting (IPTW) using the propensity score to estimate causal treatment effects in observational studies. *Stat Med.* 2015;34(28):3661-3679.
10. Austin PC. Balance diagnostics for comparing the distribution of baseline covariates between treatment groups in propensity-score matched samples. *Stat Med.* 2009;28(25):3083-3107.
11. Modra L, Higgins A, Vithanage R, Abeygunawardana V, Bailey M, Bellomo R. Sex differences in illness severity and mortality among adult intensive care patients: A systematic review and meta-analysis. *J Crit Care.* 2021;65:116-123.
12. Romo H, Amaral AC, Vincent JL. Effect of patient sex on intensive care unit survival. *Arch Intern Med.* 2004;164(1):61-65.
13. Wahba WM. Influence of aging on lung function--clinical significance of changes from age twenty. *Anesth Analg.* 1983;62(8):764-776.
14. Muscedere J, Waters B, Varambally A, et al. The impact of frailty on intensive care unit outcomes: a systematic review and meta-analysis. *Intensive care medicine.* 2017;43(8):1105-1122.
15. Tran DD, Groeneveld AB, van der Meulen J, Nauta JJ, Strack van Schijndel RJ, Thijs LG. Age, chronic disease, sepsis, organ system failure, and mortality in a medical intensive care unit. *Critical care medicine.* 1990;18(5):474-479.
16. O'Brien JM, Jr., Phillips GS, Ali NA, Lucarelli M, Marsh CB, Lemeshow S. Body mass index is independently associated with hospital mortality in mechanically ventilated adults with acute lung injury. *Critical care medicine.* 2006;34(3):738-744.

17. Sanaie S, Hosseini MS, Karrubi F, Iranpour A, Mahmoodpoor A. Impact of Body Mass Index on the Mortality of Critically Ill Patients Admitted to the Intensive Care Unit: An Observational Study. *Anesth Pain Med.* 2021;11(1):e108561.
18. Knaus WA, Wagner DP, Draper EA, et al. The APACHE III prognostic system. Risk prediction of hospital mortality for critically ill hospitalized adults. *Chest.* 1991;100(6):1619-1636.
19. Singer M, Deutschman CS, Seymour CW, et al. The Third International Consensus Definitions for Sepsis and Septic Shock (Sepsis-3). *Jama.* 2016;315(8):801-810.
20. Samanta S, Singh RK, Baronia AK, et al. Early pH Change Predicts Intensive Care Unit Mortality. *Indian J Crit Care Med.* 2018;22(10):697-705.
21. Allyn J, Vandroux D, Jabot J, et al. Prognosis of patients presenting extreme acidosis (pH <7) on admission to intensive care unit. *J Crit Care.* 2016;31(1):243-248.
22. Bellani G, Laffey JG, Pham T, Fan E, Investigators LS, the ETG. The LUNG SAFE study: a presentation of the prevalence of ARDS according to the Berlin Definition! *Critical care.* 2016;20:268.
23. van Buuren S, Groothuis-Oudshoorn K. mice: Multivariate Imputation by Chained Equations in R. *J Stat Softw.* 2011;45(3):1 - 67.
24. Marshall A, Altman DG, Holder RL, Royston P. Combining estimates of interest in prognostic modelling studies after multiple imputation: current practice and guidelines. *BMC Med Res Methodol.* 2009;9:57.
25. Perperoglou A, Sauerbrei W, Abrahamowicz M, Schmid M. A review of spline function procedures in R. *BMC Medical Research Methodology.* 2019;19(1):46.
